# Нейрофиброматоз 1 типа в сочетании с феохромоцитомой: описание клинического случая с кратким обзором литературы

**DOI:** 10.14341/probl13345

**Published:** 2023-09-11

**Authors:** А. Ю. Луговская, Т. А. Бритвин, Л. Е. Гуревич, И. С. Рог, Л. Н. Нефедова, И. А. Иловайская

**Affiliations:** Московский областной научно-исследовательский клинический институт им. М.Ф. Владимирского; Московский областной научно-исследовательский клинический институт им. М.Ф. Владимирского; Московский областной научно-исследовательский клинический институт им. М.Ф. Владимирского; Московский государственный университет имени М.В. Ломоносова; Московский государственный университет имени М.В. Ломоносова; Московский областной научно-исследовательский клинический институт им. М.Ф. Владимирского

**Keywords:** нейрофиброматоз 1 типа, феохромоцитома, параганглиома, артериальная гипертензия, генетика, иммуногистохимическое исследование

## Abstract

Представлено описание клинического случая нейрофиброматоза 1 типа (НФ-1) в сочетании с феохромоцитомой (ФХЦ) у мужчины моложе 40 лет без семейного анамнеза заболевания. Диагноз НФ-1 был установлен на основании 4 признаков заболевания (кожные изменения цвета «кофе с молоком», сколиоз, множественные нейрофибромы, узелки Лиша). Диагноз ФХЦ был установлен по данным значительного повышения уровня свободных метанефринов и норметанефринов суточной мочи, злокачественного КТ-фенотип опухоли правого надпочечника и подтвержден при патоморфологическом исследовании. В ходе генетического анализа была обнаружена новая мутация в одном из аллелей гена NF1 — делеция фрагмента гена размером 566 п.н., включающего 19 экзон размером 73 п.н. Мутация приводит к сплайсингу 18 и 20 экзонов, сдвигу рамки считывания и обрыву синтеза белка. Проведено также исследование уровня транскрипции генов RET, TMEM127, MAX, FGFR, MET, MERTK, BRAF, NGFR, Pi3, AKT, MTOR, KRAS, MAPK, ассоциированных с феохромоцитомой; обнаружено статистически значимое снижение уровня транскрипции генов KRAS и BRAF и статистически значимое повышение уровня транскрипции гена TMEM127 в сравнении с контрольными образцами, что дает возможность отнести феохромоцитому в данном случае ко 2-му кластеру генетических аномалий при параганглиомах. Данный случай свидетельствует о необходимости своевременного распознавания НФ-1 для выработки тактики дальнейшего наблюдения за пациентом и демонстрирует эффективность мультидисциплинарного подхода к диагностике и лечению ассоциированных с НФ-1 катехоламин-секретирующих опухолей.

## Abstract (en)

We presented the clinical case of neurofibromatosis type 1 (NF-1) associated with pheochromocytoma (PHEO) in a man under 40 years old without family history. The diagnosis of NF-1 was established based on 4 signs of the disease (multiple café au lait macules, scoliotic changes in posture, the presence of multiple neurofibromas, Lisch nodules). The diagnosis of PHEO was determined by a significant increase of free metanephrin/normethanephrin levels in daily urine, a malignant CT phenotype of the right adrenal tumor, and confirmed by pathomorphological study. Genetic tests revealed a new mutation in one of the alleles of NF1 gene, a deletion of a 566 bp gene fragment, including exon 19 with a size of 73 bp. This mutation leads to splicing of exons 18 and 20, frameshift, and termination of protein synthesis. A study of the level of transcription of the genes associated with PHEO (RET, TMEM127, MAX, FGFR, MET, MERTK, BRAF, NGFR, Pi3, AKT, MTOR, KRAS, MAPK) was conducted, a statistically significant decrease in the level of transcription of the KRAS and BRAF genes and increase in the level of transcription of the TMEM127 gene in comparison with control samples have been detected. This case demonstrates the need for timely recognition of NF-1 for further appropriate patient’s follow up and show the effectiveness of a multidisciplinary approach to the diagnosis and treatment of NF-1-associated catecholamine-secreting tumors.

## АКТУАЛЬНОСТЬ

Нейрофиброматозы — группа наследственных моногенных заболеваний с аутосомно-доминантным типом наследования. Основным клиническим проявлением нейрофиброматоза является формирование множественных нейрофибром — опухолей в тканях нейроэктодермального происхождения. Данная группа заболеваний включает 3 нозологии, имеющие различную генетическую природу: нейрофиброматоз I типа, нейрофиброматоз II типа и шванноматоз, которые различаются по спектру клинических проявлений, возрасту манифестации, тяжести течения и прогнозу [1]. Нейрофиброматоз I типа связан с мутациями в гене NF1 [2]. Нейрофиброматоз II типа связан с мутациями в гене NF2, кодирующем белок-супрессор опухолей мерлин, а шванноматоз — с мутациями в генах SMARCB1 и LZTR [3][4].

Нейрофиброматоз I типа (НФ-1) был впервые описан Фредериком фон Реклингхаузеном в 1882 г. в его статье «О множественных фибромах кожи и их связи с множественными невромами», поэтому также известен как болезнь фон Реклингхаузена [5]. Распространенность НФ-1 составляет 1:2500–1:4000 человек и не зависит от этнической и расовой принадлежности [6][7]. К характерным клиническим проявлениям относят пигментные кожные пятна цвета «кофе с молоком», различные по размеру, и локализации нейрофибромы, дисплазию костной ткани, узелки Лиша на радужной оболочке глаза, глиому зрительного тракта, склонность к развитию новообразований любых тканей и органов (табл. 1) [8][9]. Пациенты могут проявлять крайнюю вариабельность клинических признаков даже у лиц из одной семьи. У некоторых людей может наблюдаться сегментарный НФ-1, ограниченный одним сегментом тела, который возникает из-за соматической мутации в NF1 в период внутриутробного развития [10]. Диагноз НФ-1 верифицируется при наличии двух клинических признаков и более у пациентов без семейного анамнеза или при наличии одного критерия у пациента с семейным анамнезом НФ-1 [8–11].

**Table:** Таблица1. Диагностические критерии нейрофиброматоза-1 (НФ-1) Table 1. Diagnostic criteria for neurofibromatosis-1 (NF-1) * Критерии консенсуса NIH (1987 г.)** Критерии Legius (2021 г.)NIH — National Institutes of Health; НФ — нейрофибромы; НФ-1 — нейрофиброматоз 1 типа.

А. Без семейного анамнеза: необходимо наличие не менее двух критериев
6 и более пятен на коже цвета «кофе с молоком» (0,5 см у детей или 1,5 см у взрослых)*
2 и более кожных/внутрикожных НФ или одна плексиформная НФ*
Гиперпигментация в подмышечной и паховой областях*
Глиома зрительного нерва (хиазмы зрительного тракта)*
2 и более узелков Лиша (пигментированные гамартомы радужной оболочки глаза)*
Выраженные костные аномалии в виде дисплазии основной кости, истончения кортикального слоя длинных костей с псевдоартрозом или без такового*
Гетерозиготный патогенный вариант в гене NF1**
В. При наличии семейного анамнеза: достаточно 1 критерия

Ожидаемая продолжительность жизни снижается в среднем на 8–15 лет, при этом основной причиной смерти в возрасте до 30 лет являются злокачественные новообразования нервной ткани [2][7][12]. Неоплазии различной локализации, которые могут встречаться у пациентов с НФ-1, включают гастроэнтеропанкреатические нейроэндокринные опухоли и опухоли различных эндокринных органов, в том числе катехоламин-секретирующие опухоли (феохромоцитомы/параганглиомы — ФХЦ/ПГ) [8][13–16].

По данным литературы, распространенность ФХЦ/ПГ при НФ-1 значительно варьирует от 0,1 до 14,6% [8][17–19]. В исследовании Gruber L.M. et al. (2017 г.) проводился анализ данных литературы с 1972 до 2012 гг., согласно которому частота ФХЦ/ПГ при НФ-1 составляла от 0,11 до 1,6% [20]. Авторы провели собственное исследование, и среди общей когорты из 1415 пациентов с НФ-1, обратившихся в Mayo Clinic (США) с 1959 по 2015 гг., верифицировали 41 случай ФХЦ/ПГ, что составило 2,9%. Однако при активном тестировании подгруппы из 351 пациента было выявлено 23 случая ФХЦ/ПГ, что составляет уже 6,6% [20]. Эти данные сравнимы с результатами другого проспективного исследования Képénékian L. et al. (2016 г.), в ходе которого исключение ФХЦ/ПГ проводилось всем 156 пациентам с верифицированным диагнозом НФ-1, и частота выявления ФХЦ составила 7,7% (12 случаев из 156 пациентов) [17]. В другом проспективном исследовании, проведенном Zinnamosca L. et al. (2010 г.), распространенность ФХЦ/ПГ была еще выше и составила 14,6% (7 случаев из 48 пациентов) [18]. Таким образом, частота выявления ФХЦ/ПГ при НФ-1 зависит от типа исследований, и в проспективных исследованиях с активным скринингом катехоламин-секретирующих опухолей составляет 6,6–14,6%, что в 2–3 раза выше по сравнению с ретроспективными.

Наиболее часто (84% случаев) у пациентов с НФ-1 встречались односторонние параганглиомы надпочечниковой локализации (феохромоцитомы), двусторонние и вненадпочечниковые параганглиомы наблюдались гораздо реже (менее 10% случаев); метастатические ФХЦ при этом синдроме встречались в 11,5% [10][21][22].

Возраст пациентов с НФ-1 на момент выявления ФХЦ/ПГ существенно варьирует — от 1,5 до 74 лет, однако медиана составляет 41–42 года [19][20]. ФХЦ/ПГ может иметь весьма различные клинические проявления, основным из которых является артериальная гипертензия [23]. Однако далеко не у всех пациентов с НФ-1 отмечается повышение артериального давления. По данным крупного ретроспективного когортного исследования Gruber L.M. et al., АГ была верифицирована всего у 16 из 41 (39%) пациента с ФХЦ при НФ-1 [20], что может свидетельствовать о необходимости исключения ФХЦ при НФ-1 вне зависимости от повышенного АД [24].

Помимо процитированных немногочисленных когортных исследований пациентов с НФ-1 и ФХЦ/ПГ [11][17][18][20], в научной базе данных PubMed, начиная с 70-х гг., описано не более 80 клинических случаев. В национальной библиографической базе данных научного цитирования РИНЦ на данный момент представлено всего несколько случаев ФХЦ у пациента с НФ-1 [25–28]. Поэтому каждый новый пациент с НФ-1 и ФХЦ представляет как практический, так и научный интерес. Мы хотим продемонстрировать клинический случай НФ-1 в сочетании с ФХЦ у мужчины моложе 40 лет.

## ОПИСАНИЕ СЛУЧАЯ

Пациент Б. в возрасте 36 лет обратился к эндокринологу в клинико-диагностический центр ГБУЗ МО МОНИКИ со случайно выявленными образованиями в обоих надпочечниках по данным УЗИ органов брюшной полости. Предъявлял жалобы на периодическое повышение артериального давления (АД) до 200/120 мм рт.ст., сопровождавшееся головными болями и учащенным сердцебиением.

Из анамнеза известно, что повышение АД пациент отмечал в течение 10 лет. Наблюдался у терапевта по месту жительства, однако антигипертензивную терапию регулярно не получал. На момент обращения симптоматически принимал каптоприл 50 мг.

При физикальном обследовании выявлены множественные пигментные пятна цвета «кофе с молоком» на коже туловища и конечностей, нейрофибромы размерами от 1,5 до 10 см различной локализации (рис. 1 a–d) и изменение осанки по типу сколиоза. Рост — 172 см, вес — 73 кг, ИМТ — 24,7 кг/м². Частота сердечных сокращений (ЧСС) соответствовала пульсу и составляла 78 уд. в мин., АД в среднем составляло 160 и 100 мм рт.ст.

**Figure:**
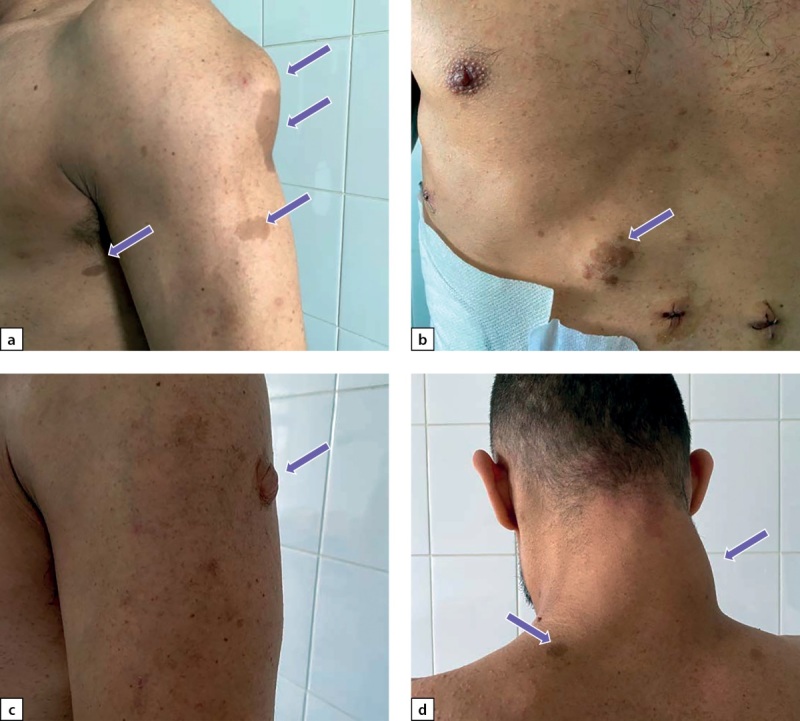
Рисунок 1. а) нейрофиброма плеча, множественные кофейные пятна; b) нейрофиброма с кофейным пятном в области живота; с) нейрофиброма плеча; d) нейрофиброма шеи, кофейные пятна. Figure 1. a) neurofibroma of the shoulder, multiple coffee stains; b) neurofibroma with coffee stain in the abdominal area; c) neurofibroma of the shoulder; d) neurofibroma of the neck, coffee stains.

При детальном расспросе выяснилось, что с раннего детства у пациента отмечалось наличие на коже пятен цвета «кофе с молоком» и нарушение осанки по типу сколиоза. В возрасте 13 лет начали появляться плотные безболезненные выпячивания по всей поверхности тела, одно из которых постепенно увеличилось до 14 см (в области правого плеча). В возрасте 26 лет была проведена операция по удалению данного образования, по данным гистологического исследования была верифицирована нейрофиброма. Однако на дополнительное обследование пациент направлен не был.

Кроме того, у 10-летней дочери пациента также имеются пятна кофейного цвета на поверхности тела. У пациента также есть два родных брата, у которых нет никаких визуальных проявлений нейрофиброматоза.

Данные обследования. В представленных лабораторных анализах (клинический анализ крови, общий анализ мочи, коагулограмма) показатели были в пределах референсных значений.

По данным биохимического анализа крови, было выявлено повышение глюкозы крови натощак до 6,5 ммоль/л.

По данным мультиспиральной компьютерной томографии (МСКТ) брюшной полости и забрюшинного пространства с в/в контрастным усилением: в правом надпочечнике, занимая все его пространство, определяется объемное образование с четкими контурами, размерами 80х75х85 мм, плотностью в артериальную фазу 66 НU, в венозную фазу 69 НU, в паренхиматозную фазу 62 НU, в отсроченную до 51 НU (рис. 2 a, b). Кроме того, в забрюшинном пространстве, слева, с деформацией левой почки на уровне средней трети, определяется объемное образование размерами 49х37х47 мм, преимущественно мягкотканой плотности 31 НU, неоднородной структуры, с наличием участков пониженной плотности. Накопление контрастного вещества в артериальную фазу 86 НU, в венозную фазу 112 НU, в паренхиматозную фазу 85 НU, в отсроченную до 62 НU (рис. 3 a, b).

**Figure:**
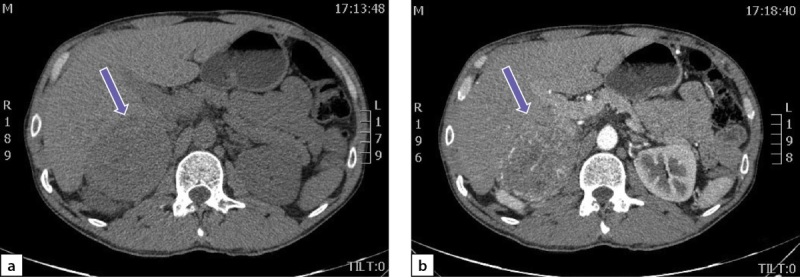
Рисунок 2. Феохромоцитома правого надпочечника:а) нативная фаза; b) после введения контрастного препарата. Figure 2. Pheochromocytoma of the right adrenal gland: a) native phase; b) after administration of a contrast agent.

**Figure:**
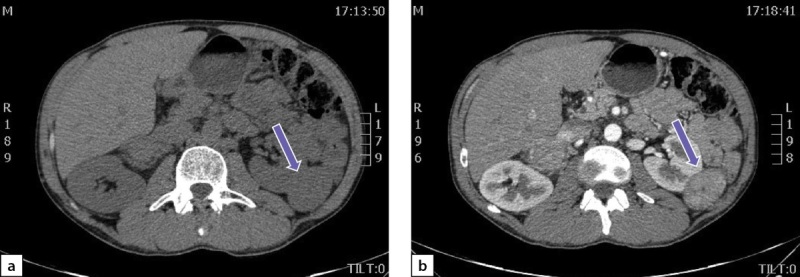
Рисунок 3. Образование забрюшинного пространства слева:а) нативная фаза; b) после введения контрастного препарата. Figure 3. Formation of the retroperitoneal space on the left: a) native phase; b) after administration of a contrast agent

Учитывая молодой возраст пациента, злокачественный КТ-фенотип выявленных образований и наличие артериальной гипертензии, у пациента были все основания для исключения ФХЦ.

По данным гормонального обследования (табл. 2) выявлено значительное повышение метанефринов и норметанефринов суточной мочи.

**Table:** Таблица 2. Лабораторные исследования Table 2. Laboratory tests

Показатели	До лечения	Референсные значения
Метанефрины суточной мочи, мкг/сут	3105	2,9–52,9
Норметанефрины суточной мочи, мкг/сут	3723	5,7–67,7
Кортизол сут. мочи, нмоль/л	390	101,2–535,7
Альдостерон крови, пг/мл	234	25,2–392
Ренин крови, мкМЕд/мл	43,5	4,4-46,1

Для оценки функционального состояния сердечно-сосудистой системы выполнены ЭКГ, суточный мониторинг АД и эхокардиография. Отмечена синусовая тахикардия до 83 в мин., подтверждена стойкая систоло-диастолическая артериальная гипертензия (АГ) со средними значениями систолического АД 153 мм рт.ст. и диастолического АД 103 мм рт.ст. в дневное время и систолического АД 154 мм рт.ст. и диастолического АД 100 мм рт.ст. в ночное время, также выявлена умеренная гипертрофия миокарда левого желудочка.

По результатам биомикроскопии обоих глаз, выявлены множественные глыбки пигмента (узелки Лиша) по всей поверхности радужки (рис. 4).

**Figure:**
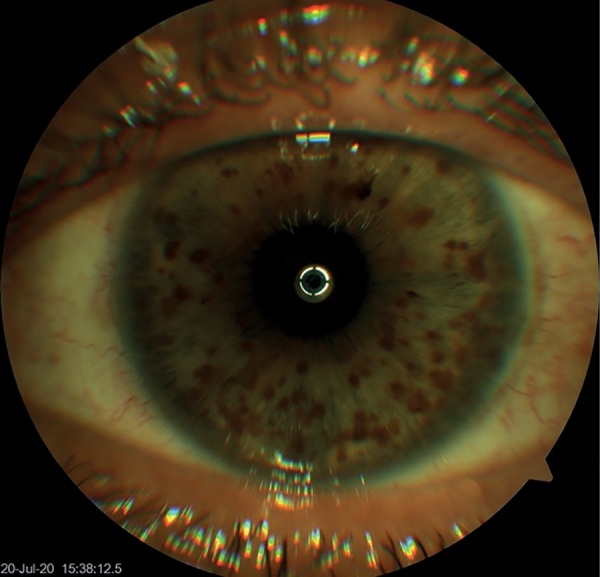
Рисунок 4. Множественные узелки Лиша на роговице глаза. Figure 4. Multiple Lisch nodules on the cornea of the eye

Таким образом, по данным осмотра, у пациента было отмечено 4 клинических признака НФ-1. Также в ходе лабораторно-инструментального обследования подтверждено наличие катехоламин-продуцирующей опухоли.

Клинический диагноз: нейрофиброматоз 1 типа. Феохромоцитома правого надпочечника pT2N0M0. Образование забрюшинного пространства слева.

Опухоль правого надпочечника имела бóльшие размеры и более высокие плотностные характеристики по данным МСКТ, поэтому первым этапом хирургического лечения было решено провести удаление правого надпочечника с опухолью. После предоперационной подготовки доксазозином в суточной дозе 4 мг и бисопрололом 5 мг пациенту была выполнена лапароскопическая адреналэктомия с опухолью справа.

По данным гистологического исследования: опухоль правого надпочечника солидного строения из полиморфных, преимущественно из светлых клеток с онкоцитарной морфологией с широкой эозинофильной гранулированной цитоплазмой, умеренным ядерным полиморфизмом (рис. 5 a, b). Митозы, некрозы, инвазия сосудов не обнаружены. Для уточнения гистогенеза опухоли и ее клинического прогноза было выполнено ИГХ-исследование: клетки опухоли интенсивно диффузно экспрессировали синаптофизин и хромогранин А (рис. 5 c). Экспрессия протеина S-100 была неравномерной и выявлялась очагово в цитоплазме и ядрах опухолевых клеток и только в единичных сустантекулярных (поддерживающих) клетках (рис. 5 d, e). Экспрессия виментина также была очаговой, неравномерной, индекс пролиферации Ki-67 был в пределах 1,5% (рис. 5 f). Заключительный патоморфологический диагноз: «Феохромоцитома правого надпочечника, онкоцитарный вариант».

**Figure:**
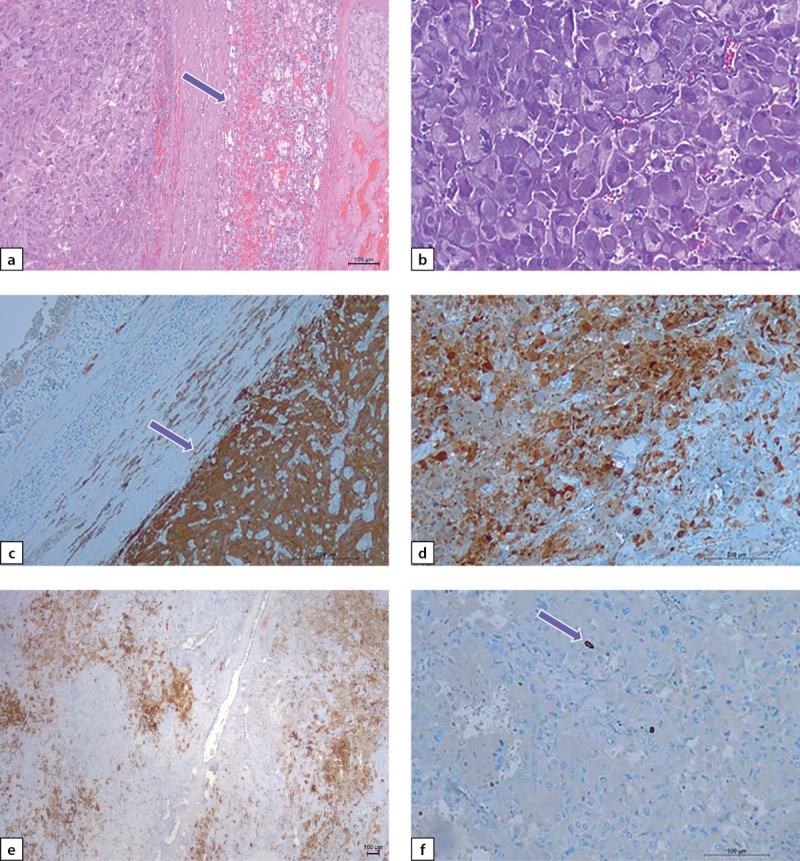
Рисунок 5 a–f. Феохромоцитома правого надпочечника.a) опухоль от окружающей коры надпочечника (показано стрелкой) отделена широкой капсулой; b) онкоцитарная морфология опухоли: клетки с обильно гранулированной эозинофильной цитоплазмой; c) экспрессия синптофизина в клетках опухоли (показано стрелкой); d, e) неравномерная экспрессия протеина S100 в опухоли, преимущественно в цитоплазме и ядрах и только в отдельных вытянутых сустантекулярных клетках; f) единичные пролиферирующие клетки опухоли с ядрами, мечеными Ki-67 (показано стрелкой); a, b — окраска гематоксилином и эозином; c–f: иммуногистохимическая реакция. Увеличение: a, c, e — х125; b — х400; e, f — х250. Figure 5 a–f. Pheochromocytoma of the right adrenal gland.

Через 6 месяцев после хирургического лечения отмечена нормализация АД без антигипертензивных препаратов. Уровни производных катехоламинов в суточной моче существенно снизились, однако полностью не нормализовались: метанефрины — 115 мкг/сут (2,9–52), норметанефрины — 208 мкг/сут (5,7–67). По данным МСКТ брюшной полости и забрюшинного пространства с в/в контрастным усилением, в забрюшинном пространстве слева визуализируется объемное образование размерами 52х38х49 мм (ранее 49х37х47мм), преимущественно мягкотканой плотности (31 НU), неоднородной структуры, с наличием участков пониженной плотности, которое деформирует левую почку на уровне средней трети; накопление контрастного препарата в артериальную фазу 86 НU, в венозную фазу 112 НU, в паренхиматозную фазу 85 НU, в отсроченную до 62 НU (рис. 6).

**Figure:**
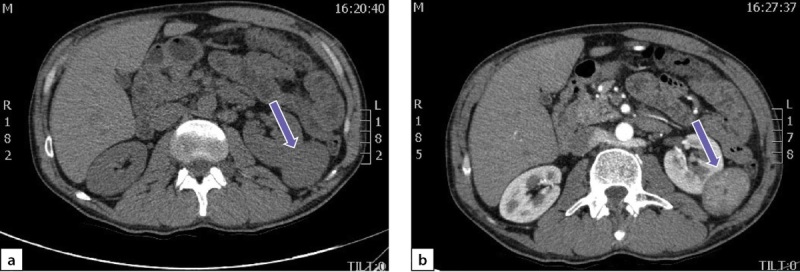
Рисунок 6. Образование забрюшинного пространства слева: а) нативная фаза; b) после введения контрастного препарата. Figure 6. Formation of the retroperitoneal space on the left: a) native phase; b) after administration of a contrast agent.

Следующим этапом лечения выполнено лапароскопическое удаление опухоли забрюшинного пространства слева (макропрепарат представлен на рис. 7). При гистологическом исследовании ограниченная четкой капсулой, на большом протяжении образованная переплетающимися пучками и закручивающимися в виде клубков веретеновидными клетками (рис. 8 a, b). При ИГХ-исследовании клетки опухоли экспрессировали виментин, неравномерно и очагово — протеин S100 и нейрофиламенты и не экспрессировали NSE и хромогранин А. Морфологически была верифицирована нейрофиброма забрюшинного пространства.

**Figure:**
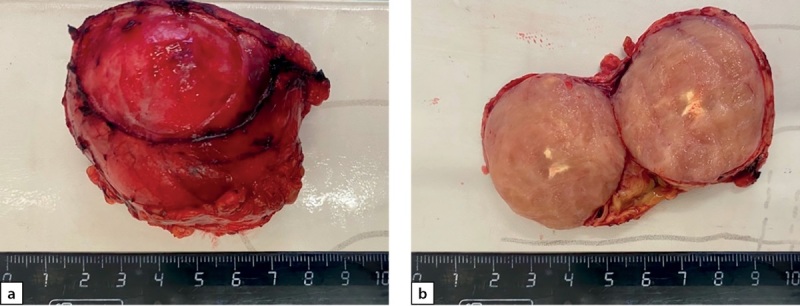
Рисунок 7 a–b. Опухоль забрюшинного пространства. Макропрепарат. b. Образование в разрезе. Figure 7 a–b. Tumor of the retroperitoneum. Macropreparation. b. Education in context.

**Figure:**
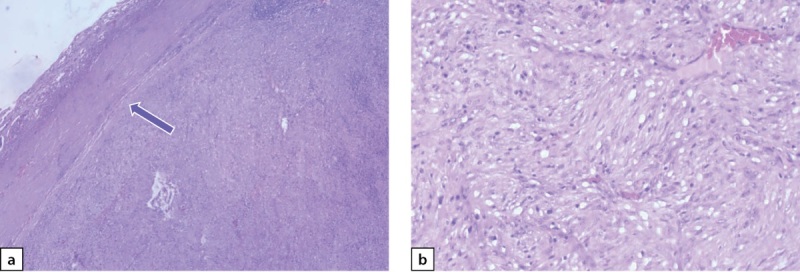
Рисунок 8 a–b. Нейрофиброма забрюшинного пространства слева.a) опухоль отделена от брюшной стенки (показано стрелкой); b) переплетающиеся пучки и клубки из веретеновидных клеток. Окраска гематоксилином и эозином. Увеличение: a — х40. Figure 8 a–b. Neurofibroma of the retroperitoneal space on the left.

Пациенту проведен молекулярно-генетический анализ: выполнено секвенирование экзонов гена NF1 по Сэнгеру, которое обнаружило не описанную ранее в базе данных ClinVar (https://www.ncbi.nlm.nih.gov/clinvar) делецию в позиции гена с 133703 п. н. по 134267 п. н. (GeneBank ID AY796305). Данный участок гена содержит 19 экзон размером 73 п. н., выпадение которого должно приводить к сдвигу рамки считывания и обрыву синтеза белка. Сдвиг рамки считывания был подтвержден при анализе транскрипта методом обратной транскрипции на мРНК пациента и ПЦР с паймерами, фланкирующими 18 и 20 экзоны. Сдвиг рамки считывания приводит к «выключению» функциональной активности всех основных доменов белка. На рис. 9 представлена схема строения гена с указанием позиции делеции.

**Figure:**
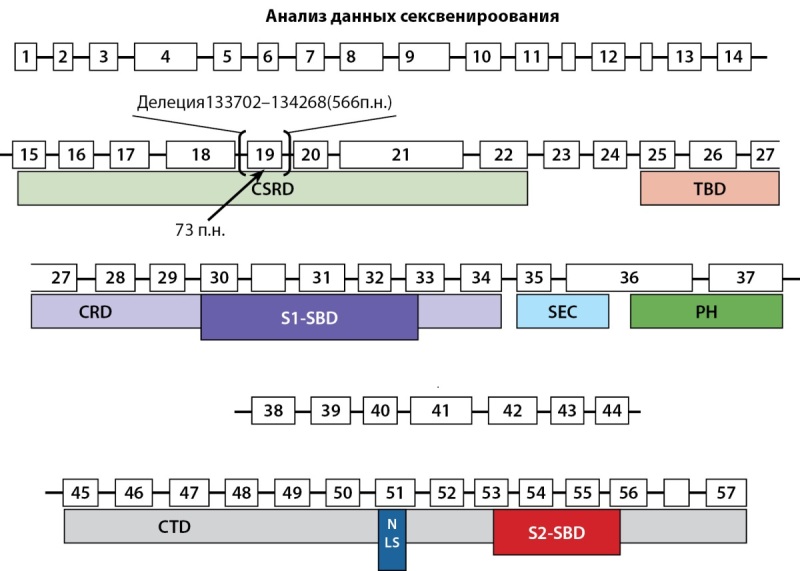
Рисунок 9. Схема строения гена NF1. Пронумерованными прямоугольниками показаны экзоны гена. Под ними обозначены домены белка. Показана позиция делеции фрагмента гена размером 566 п.н. и экзона 19 размером 73 п.н. Figure 9. Scheme of the structure of the NF1 gene.

Для оценки экспрессии генов, ассоциированных с ФХЦ/ПГ и сигнальными путями, в которых задействован нейрофибромин (рис. 10), было произведено исследование уровня транскрипции генов RET, TMEM127, MAX, FGFR, MET, MERTK, BRAF, NGFR, Pi3, AKT, MTOR, KRAS, MAPK.

**Figure:**
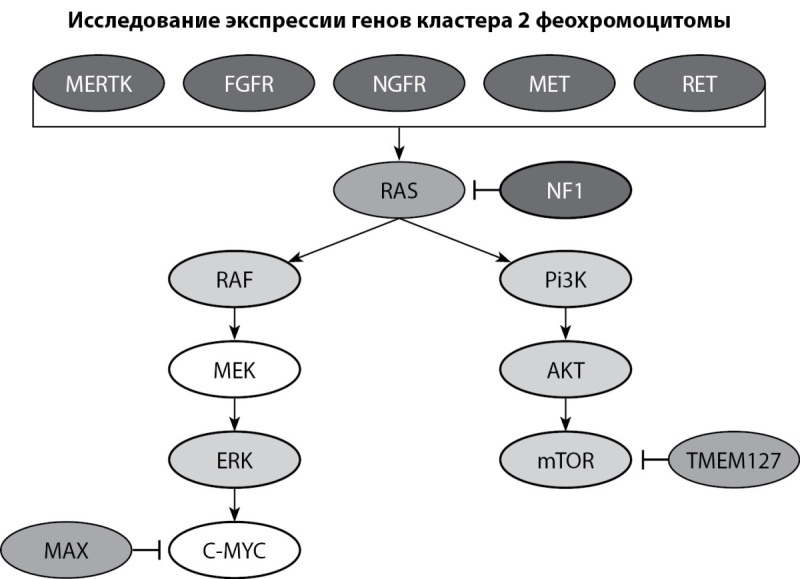
Рисунок 10. Кластер генов, ассоциированных с ФХЦ/ПГ и НФ-1, экспрессия которых была исследована у пациента с НФ-1. Figure 10. Cluster of genes associated with FCC/PG and NF-1, the expression of which was studied in a patient with NF-1. Обозначения: ↓ — активация гена, ⊥ — ингибирование гена.

Для изучения уровня экспрессии генов были поставлены полимеразные цепные реакции на матрице кДНК пациента и контролей методом ПЦР в реальном времени (рис. 11). Измеряли относительный уровень транскрипции генов путем нормирования уровня транскрипции исследуемых генов на уровни транскрипции референсных генов (Agtr1, ACTB) при помощи метода ΔСt. Выборки состояли из 5 образцов для каждого гена. Для определения статистической значимости различий переменных двух независимых групп использовали U-тест Манна-Уитни.

**Figure:**
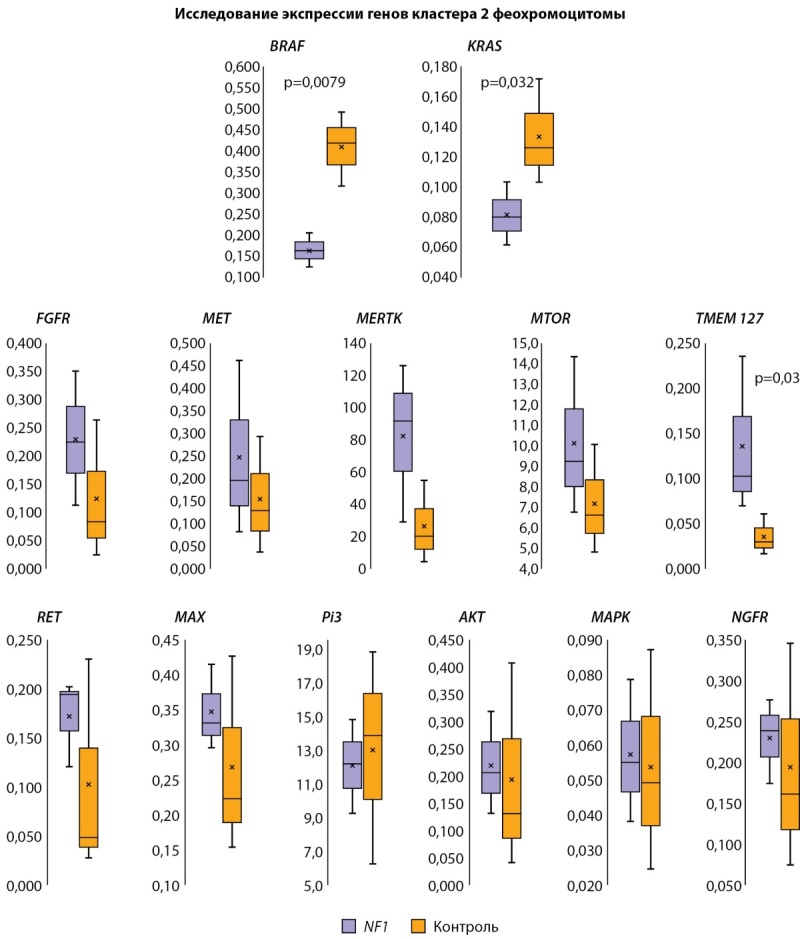
Рисунок 11. Уровень транскрипции генов RET, TMEM127, MAX, FGFR, MET, MERTK, BRAF, NGFR, Pi3, AKT, MTOR, KRAS, MAPK у пациента с НФ-1 в сравнении с контролем. По оси ординат — уровень относительной транскрипции генов. Figure 11. Transcription level of the genes RET, TMEM127, MAX, FGFR, MET, MERTK, BRAF, NGFR, Pi3, AKT, MTOR, KRAS, MAPK in a patient with NF-1 compared to the control. The y-axis is the level of relative gene transcription. Обозначения: NF1 — пациент с НФ-1.

Так как нейрофибромин является ингибитором сигнального пути RAS, который активирует каскады RAF/MEK/ERK и mTOR, мы ожидали увидеть увеличение уровня транскрипции генов-участников данных сигнальных путей: RET, TMEM127, MAX, FGFR, MET, MERTK, NGFR, Pi3, AKT, MTOR, MAPK, BRAF и KRAS у пациента с НФ-1. Данная тенденция подтвердилась для генов RET, TMEM127, MAX, FGFR, MET, MERTK, NGFR, Pi3, AKT, MTOR, MAPK, но статистический анализ не подтвердил достоверность различий для большинства генов. Статистически значимым оказалось снижение уровня транскрипции генов BRAF и KRAS и увеличение уровня транскрипции гена TMEM127.

Таким образом, для подтверждения и интерпретации данного результата необходимо проведение дальнейшего исследования с расширенной выборкой пациентов и контрольной группой.

В течение последующих двух лет, при динамическом наблюдении, пациент жалоб не предъявлял, при измерении АД повышения не фиксировалось (без антигипертензивной терапии). По результатам лабораторных и инструментальных исследований данных за наличие рецидива ФХЦ не получено.

## ОБСУЖДЕНИЕ

В представленном клиническом случае у пациента с подросткового возраста имелись фенотипические признаки НФ-1: по данным осмотра, у пациента сразу было отмечено наличие 3 классических признаков НФ-1 (множественные пигментные пятна цвета «кофе с молоком», сколиотическое изменение осанки, наличие множественных нейрофибром), что уже позволяет поставить диагноз «НФ» [11][17][29]. Результаты проведенной биомикроскопии обоих глаз дополнили диагноз НФ-1. Однако на разных этапах наблюдения за пациентом на эти признаки не было обращено должного внимания.

В существующих клинических рекомендациях по диагностике и лечению НФ-1 нет указания о необходимости проведении рутинного скрининга на ФХЦ/ПГ при НФ-1 [11]. Тем не менее необходимо отметить, что клинические рекомендации датированы 2007 г., в то время как более поздние исследования, показавшие высокую частоту ФХЦ/ПГ при НФ-1, были проведены позже. По мнению Képénékian L. et al., скрининг ФХЦ/ПГ рекомендовано проводить всем пациентам с НФ-1 старше 40 лет [17], в то время как Gruber L.M. et al. рекомендуют проведение биохимических тестов на выявление ФХЦ/ПГ у пациентов любого возраста в момент диагностики НФ-1 и затем каждые 3 года в течение всей жизни [17][20]. В нашем случае у пациента с клиническими признаками НФ-1 повышение АД фиксировалось с 26 лет (т.е. моложе 40 лет). Однако поводом для исключения гиперкатехоламинемии стали случайно выявленные образования правого надпочечника и забрюшинного пространства слева в возрасте 36 лет. По данным МСКТ, образования были сходной высокой нативной плотностью (>30 НU) и значительных размеров (>4 см), что соответствовало злокачественному КТ-фенотипу и было патогномонично для ФХЦ/ПГ [30][31]. Данные лабораторного обследования однозначно подтвердили наличие ФХЦ/ПГ у нашего пациента с НФ-1, но нельзя было сделать однозначного заключения в отношении топического диагноза. Образование правого надпочечника имело большие размеры, «худшие» параметры нативной плотности и процента вымывания контрастного вещества, поэтому первым этапом была произведена лапароскопическая адреналэктомия с опухолью справа, гистологически верифицирована ФХЦ. После хирургического лечения у пациента спонтанно нормализовалось АД без антигипертензивной терапии, значительно снизились (однако полностью не нормализовались) уровни метанефринов и норметанефринов.

Образование забрюшинного пространства слева, по данным МСКТ, не исходило из надпочечника, однако высокие плотностные характеристики, отсутствие полной нормализации уровней метанефринов после удаления феохромоцитомы справа и прогрессирующий рост не позволили полностью исключить параганглиому. Кроме того, признаки сдавления левой почки и значительные размеры образования являлись самостоятельными показаниями для хирургического лечения. В связи с этим вторым этапом было произведено лапароскопическое удаление образования забрюшинного пространства слева, которое гистологически было верифицировано как нейрофиброма забрюшинного пространства. Уровни свободных метанефринов и норметанефринов в суточной моче полностью нормализовались после второй операции. Было высказано предположение, что некоторое повышение уровня метанефринов и норметанефринов, сохранявшееся после первой операции, могло быть связано со значительным сдавлением левого надпочечника нейрофибромой.

Сложность молекулярного тестирования для выявления мутаций в NF1 связана с большим размером гена (около 60 экзонов) и большим разнообразием патологических мутаций. В настоящее время около половины выявленных мутаций гена NF1 уникальны, и пока не установлены четкие закономерности между локализацией мутаций и клинической картиной заболевания, частотой возникновения определенных вариантов опухолей и прогнозом клинического течения заболевания у пациентов с этим синдромом. Требуется многоэтапный подход с анализом геномной ДНК и мРНК крови для обнаружения целых делеций в гене NF1. В нашем случае у пациента был генетически подтвержден диагноз НФ-1, впервые выявлена делеция в 19 экзоне A 136432 со сдвигом рамки считывания гена NF1, не описанная ранее. К сожалению, по ряду причин не удалось обследовать семью пациента, включая дочь с клиническими признаками НФ-1.

Помимо мутации гена NF1, были зафиксированы комплексные изменения активности других генов (RET, FGFR, TMEM127, MAX, MET, MERTK, NGFR, Pi3, AKT, MTOR, MAPK). Для выявления причины предполагаемого неравномерного изменения экспрессии генов кластера 2 ФХЦ, связанной с мутацией в гене NF1, необходимо проведение дальнейших исследований.

В настоящее время все ФХЦ/ПГ считаются злокачественными новообразованиями, их своевременная диагностика и лечение позволит улучшить качество жизни с возможным ее продлением [32]. Прогрессирование ФХЦ/ПГ в исследовании Gruber L.M. et al. (2017 г.) [20] за период с 1959 по 2015 гг. зафиксировано у 7,3% из 41 больного, в то время как в исследовании Képénékian L. et al. (2016 г.) прогрессирования заболевания выявлено не было, автор связывает это с коротким сроком наблюдения (20 месяцев) [17]. В нашем случае длительность наблюдения за пациентом составляет 26 месяцев, прогрессирование ФХЦ не отмечено. Гены, в которых выявлены изменения экспрессии у нашего пациента, относятся ко 2 (киназно-сигнальному) кластеру генетических нарушений, ассоциированных с развитием ФХЦ/ПГ [33]. Для опухолей, возникающих вследствие мутаций данного кластера, характерны низкий метастатический потенциал, что дает надежду на безрецидивное течение заболевания в данном случае.

## ЗАКЛЮЧЕНИЕ

Нейрофиброматоз 1 типа достаточно редкое, однако узнаваемое заболевание, диагностика которого базируется на клинических критериях и не требует обширного лабораторно-инструментального обследования. Мы представляем пациента с пропущенным диагнозом НФ-1, несмотря на характерные клинические признаки заболевания с подросткового возраста. Пациенты с НФ-1 нуждаются в длительном наблюдении для раннего выявления возможных ассоциированных злокачественных образований, среди которых — ФХЦ/ПГ. При наличии НФ-1 частота выявления ФХЦ/ПГ может достигать 14,6%, что чаще, чем в общей популяции. По мнению ряда авторов, наличие НФ-1 в любом возрасте уже является основанием для исключения ФХЦ/ПГ, вне зависимости от наличия артериальной гипертензии. Отсутствие верификации диагноза НФ-1 привело и к отсутствию настороженности в отношении ФХЦ/ПГ, несмотря на артериальную гипертензию. КТ-фенотип ФХЦ/ПГ правого надпочечника оказался схожим с КТ-фенотипом нейрофибромы забрюшинного пространства, что может затруднять топическую диагностику; однако детальное патоморфологическое исследование позволило провести дифференциальный диагноз. В ходе генетического анализа была обнаружена новая гетерозиготная мутация гена NF1 и изменение экспрессии генов, ассоциированных с киназно-сигнальным кластером развития ФХЦ/ПГ.

Данный случай свидетельствует о необходимости своевременного распознавания НФ-1 для выработки тактики дальнейшего наблюдения за пациентом и демонстрирует эффективность мультидисциплинарного подхода к диагностике и лечению ассоциированных с НФ-1 катехоламин-секретирующих опухолей.

## ДОПОЛНИТЕЛЬНАЯ ИНФОРМАЦИЯ

Источники финансирования. Работа выполнена при частичной поддержке Программы развития МГУ, проект № 23-Ш04-34.

Конфликт интересов. Авторы декларируют отсутствие явных и потенциальных конфликтов интересов, связанных с содержанием настоящей статьи.

Участие авторов. Все авторы одобрили финальную версию статьи перед публикацией, выразили согласие нести ответственность за все аспекты работы, подразумевающую надлежащее изучение и решение вопросов, связанных с точностью или добросовестностью любой части работы.

Согласие пациента. Авторы настоящей статьи получили письменное разрешение от упоминаемых в статье пациентов на публикацию их медицинских данных в журнале «Проблемы эндокринологии».
